# Mutations in Splicing Factor Genes in Myeloid Malignancies: Significance and Impact on Clinical Features

**DOI:** 10.3390/cancers11121844

**Published:** 2019-11-22

**Authors:** Valeria Visconte, Megan O. Nakashima, Heesun J. Rogers

**Affiliations:** 1 Department of Translational Hematology and Oncology Research, Taussig Cancer Institute, Cleveland Clinic, Cleveland, OH 44195, USA; visconv@ccf.org; 2 Department of Laboratory Medicine, Cleveland Clinic, Cleveland, OH 44195, USA; NAKASHM@ccf.org

**Keywords:** splicing factor genes, mutations, AML, MDS, therapies

## Abstract

Components of the pre-messenger RNA splicing machinery are frequently mutated in myeloid malignancies. Mutations in *LUC7L2, PRPF8,* *SF3B1*, *SRSF2*, *U2AF1*, and *ZRSR2* genes occur at various frequencies ranging between 40% and 85% in different subtypes of myelodysplastic syndrome (MDS) and 5% and 10% of acute myeloid leukemia (AML) and myeloproliferative neoplasms (MPNs). In some instances, splicing factor (SF) mutations have provided diagnostic utility and information on clinical outcomes as exemplified by *SF3B1* mutations associated with increased ring sideroblasts (RS) in MDS-RS or MDS/MPN-RS with thrombocytosis. *SF3B1* mutations are associated with better survival outcomes, while *SRSF2* mutations are associated with a shorter survival time and increased AML progression, and *U2AF1* mutations with a lower remission rate and shorter survival time. Beside the presence of mutations, transcriptomics technologies have shown that one third of genes in AML patients are differentially expressed, leading to altered transcript stability, interruption of protein function, and improper translation compared to those of healthy individuals. The detection of SF mutations demonstrates the importance of splicing abnormalities in the hematopoiesis of MDS and AML patients given the fact that abnormal splicing regulates the function of several transcriptional factors (*PU.1, RUNX1,* etc.) crucial in hematopoietic function. This review provides a summary of the significance of the most frequently mutated SF genes in myeloid malignancies and an update on novel targeted therapies in experimental and clinical trial stages.

## 1. Genetics of Myeloid Malignancies

**Figure 1:**
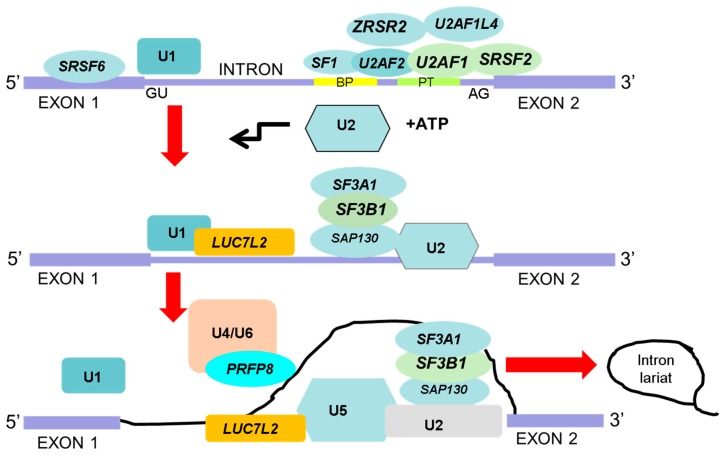
Spliceosome complex. Pre-mRNA splicing initiates with the recruitment of U1 snRNP to the 5′ splice site (SS). Serine-arginine rich (SR) proteins such as SRSF6 are localized nearby the 5′SS regulating alternative splicing. The SF1 protein and the larger subunit of the U2 auxiliary factor (U2AF), U2AF2, both gather to bind the branch point (BP) sequence and the polypyrimidine tract (PT). The smaller subunit of U2AF (U2AF1) binds to the AG dinucleotide of the 3′SS, interacting with both U2AF2 and the splicing factor SRSF2. ZRSR2 and U2AF1L4 (other two RNA-binding proteins) are recruited to interact with U2AF complex (U2AF1 and U2AF2) in order to recognize the 3′SS. After the recognition of the 3′SS, the U2 snRNP, together with SF3A1, SF3B1, and SAP130*, is recruited to the 3′SS. In the meantime, the U1 and LUC7L2 are recruited to the 5′SS and the U4/U6 and U5 complex, together with PRFP8, localize on the BP and PT regions to catalyze the release of the intron lariat and ligation of exons. *SAP130: Official gene name is *SF3B3*.

Myelodysplastic syndromes (MDS) are a genetically and clinically diverse group of clonal stem cell malignancies characterized by inefficient hematopoiesis, peripheral blood cytopenias, and an increased risk of transformation to acute myeloid leukemia (AML). Genetic alterations occurring at the level of a multipotent stem cell are believed to accelerate clonal evolution from MDS, sometimes resulting in transformation to acute leukemia, although no specific disease-initiating defect has been identified to date. In the last decade, several gene mutations acting as driver events have been identified with various frequencies across the spectrum of MDS and AML. Disease evolution is driven by positive selection of driver mutations. So far, more than 30 driver genes have been associated with the pathogenesis of leukemia [1,2]. Gene functional analysis has clustered these genes into pathways including DNA methylation, chromatin remodeling, RNA-splicing, cohesion complex, RAS family signaling, gene transcription, and DNA repair. One of the major classes of driver mutations is mutations in splicing factors (SF) (*SF3B1*, *U2AF1*, *SRSF2*, *ZRSR2*, *PRPF8*, *LUC7L2*) (Figure 1). SF genes encode for components of the spliceosomes. Spliceosomes are nuclear structures composed of five small nuclear RNAs (snRNA) and approximately 150 proteins, which catalyze the splicing reaction. The complex removes non-coding sequences (introns) from precursor messenger RNA and ligates coding sequences (exons) in order to form mature mRNA transcripts through early and late steps. Early steps involve recognition of the 5′ and 3′ exon/intron junctions and late steps recall all the spliceosome together. The information to define the splicing regions are included in short and conserved sequences at the 5′ splice site (SS), the 3′SS, and the branch site (BS). In higher eukaryotes, the BS is located approximately 18–40 nucleotides upstream of the 3′SS and is followed by a polypyrimidine tract. Many genes in humans are spliced into two or more transcripts with altered sequences through a process called alternative splicing. There are two types of pre-mRNA introns: U2-dependent (major spliceosome), which accounts for almost all the human introns, and U12-dependent (minor spliceosome), which accounts for less than a thousand introns [3,4].

As a whole, SF mutations are detected in 45–85% of different MDS subtypes and in 5–10% of primary AML [5,6]. Mutations in the SF genes occur in the same spots in MDS and AML. In MDS, SF mutations are associated with mutations in epigenetic genes, e.g., *SF3B1* with *DNMT3A* mutations, *SRSF2* with *RUNX1*, *IDH1/2* and *ASXL1* mutations and *U2AF1* with *ASXL1* and *DNMT3A* mutations. In AML, SF mutations are associated mainly with *RUNX1, ASXL1, IDH2* and *TET2* [6].

**Figure 2:**
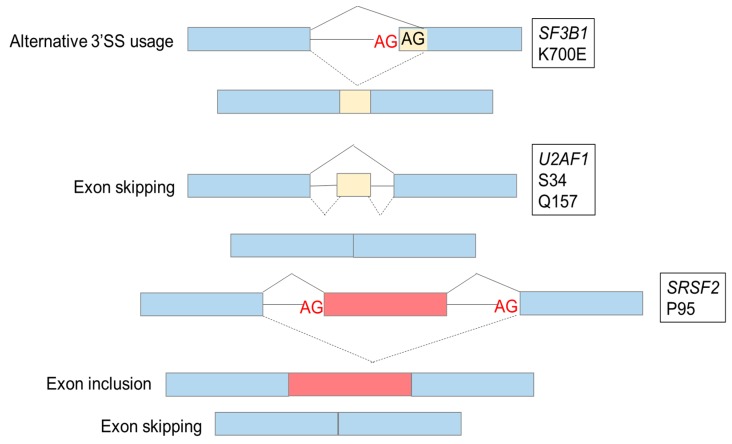
Consequences of mutations in splicing factors. Examples of consequences of splicing factors mutations are alternative 3′ splicing site (SS) usage caused by *SF3B1* mutations, increased exon skipping caused by *U2AF1* mutations, exon skipping and exon inclusion caused by *SRSF2* mutations.

The mutations often occur in genes controlling 3′SS selection. Missense mutations are a hallmark of *SF3B1*, *SRSF2*, and *U2AF1* and inactivating mutations (nonsense or frameshift) are often detected in the *ZRSR2* gene. These SF mutations are the most commonly observed mutations in MDS and AML, and are found in approximately two-thirds of SF-mutated cases. Except for *ZRSR2*, the other SF are part of the U2-type spliceosome. The heterozygous configuration of the mutations at selective sites and the absence of nonsense or truncating mutations in major SF (*SF3B1*, *SRSF2*, *U2AF1*) suggest that SF are likely gain-of-function oncogenes [7,8]. The consequences of SF mutations and the production of alternative transcripts are presented in Figure 2.

**Figure 3:**
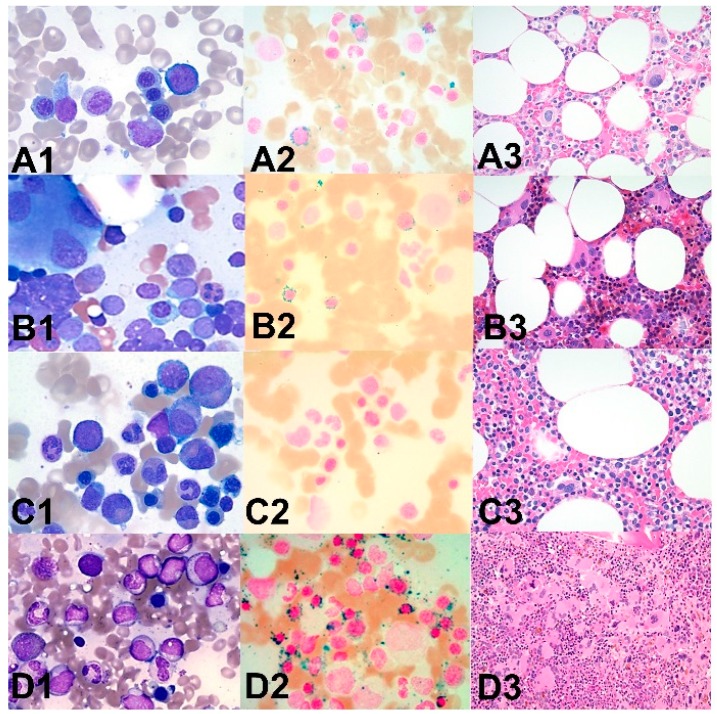
Morphologic features of representative patients with mutations in splicing factors. Bone marrow aspiration smears (1: Wright–Giemsa stain, ×500) and iron stains (2, ×400) and core biopsies (3: H&E stain, ×200) in representative cases with myeloid malignancies and mutation in splicing factor genes. A1–3: A patient with myelodysplastic syndrome with ring sideroblasts and multilineage dysplasia with *SF3B1* and *ZRSR2* mutation (detected by next generation sequencing) showing mild dyserythropoiesis (**A1**), increased ring sideroblasts (**A2**) and dysmegakaryopoiesis (**A3**). B1–3: A patient with acute myeloid leukemia with *SF3B1* mutation showing increased myeloblasts and dysmegakaryopoiesis (**B1**,**B3**) and increased ring sideroblasts (**B2**). C1–3: A patient with myelodysplastic syndrome with *U2AF1* mutation showing minimal dyserythropoiesis and no increased blasts (**C1**), no increased ring sideroblasts (**C2**) and mild dysmegakaryopoiesis (**C3**). D1–3: A patient with myelodysplastic/myeloproliferative neoplasm with thrombocytosis and ring sideroblasts with *SF3B1* mutation showing no significant dysplasia in granulocytes and erythroid cells (**D1**), increased ring sideroblasts (**D2**) and dysmegakaryopoiesis (**D3**).

Mutations in *SF3B1*, *SRSF2*, and *U2AF1* have been associated with specific disease subtypes with *SF3B1* being mostly mutated in MDS-RS, *SRSF2* occurring mostly in chronic myelomonocytic leukemia (CMML), and *U2AF1* in secondary AML [9]. Representative cases of SF mutant patients and morphologic features are presented in Figure 3.

## 2. Overview of Splicing Factor Mutations in Myeloid Malignancies

***SF3B1***. The *SF3B1* gene (chromosome 2q33.1) encodes a core component of the U2 nuclear ribonucleoprotein, which recognizes the 3′SS at intron-exon junctions. Mutations are located preferentially in four consecutive HEAT (Huntington elongation factor 3 protein phosphatase 2A, and the yeast PI3-kinase TOR1) domains of the C-terminal region, with the lysine to glutamic acid substitution at codon 700 (K700E) accounting for more than 50% of all mutant cases. Other common hotspot mutations involve the conserved amino acids 622, 625, 662, and 666 [10]. As such, all mutations occur quite distant to the region of the protein involved in the 3′ branch site recognition suggesting that these alterations do not influence the RNA-binding properties of the protein. Studies in murine models have shown that homozygous mutations of the *SF3B1* gene are incompatible with life [11,12]. Clonal analysis and in vitro experiments of human leukemia cells have shown that *SF3B1* mutations are initiating events which occur in rare lympho-myeloid hematopoietic stem cells (HSCs) (Lin− CD34+ CD38− CD90+ CD45RA−) [13,14]. The cells seem to provide a marked clonal advantage to the MDS-RS HSCs. Indeed, the percentage of RS in the bone marrow correlates with the *SF3B1* variant allele frequency [15]. Xenotransplantation studies showed that clones with *SF3B1* mutations are often not suppressed by other clones with different mutations (e.g., *DNMT3A*, *JAK2*) [14]. *SF3B1* mutations are significantly associated with older age [16]. More recently, *SF3B1* mutations have also been found in a proportion of individuals with clonal hematopoiesis of indeterminate potential (CHIP) at risk for developing malignancies including MDS [17]. Mutual exclusivity in SF mutations suggest that co-occurring mutations are incompatible with life. SF mutations induce a variety of splicing changes which are unique to specific SF. RNA-sequencing analysis of many tumor types have found that splicing abnormalities in *SF3B1* mutant cells are exclusively related to the utilization of a cryptic splice acceptor site located upstream of the canonical 3′SS [18,19]. These splicing events often produce framing errors. Among the several target genes of SF, most have been studied for effects of mutations in the *SF3B1* gene. *SF3B1* mutant cells have a defect in the splicing of ABCB7 (ATP Binding Cassette Subfamily B, Member 7) due to the usage of an alternative 3′SS which causes an early termination of the protein [20,21]. The change in splicing seems to be the major determinant in decreasing ABCB7 mRNA levels and consequent increase in iron deposition (a feature of MDS-RS). Moreover, alternative 3′SS were also observed in *PPOX* (1q23.3) and *TMEM14C* (6p24.2) genes, particularly during erythroid maturation, using in vitro cellular systems [22]. Other events of aberrant splicing with resultant low mRNA production were also found in tumor suppressor genes including *NF1*, *DICER1*, *PML*, *PDS5A*, *MAP3K7*, and *PPP2R5A* [23]. Mice conditionally expressing K700E mutation (Mx1-Cre *Sf3b1*^+/K700E^) develop progressive macrocytic anemia, inefficient cellular differentiation, and failure in bone marrow reconstitution capacity [12].

*SF3B1* mutations often co-occur with *TET2* mutations [24]. Double mutant mice generated by the breeding of *Sf3b1*^+/K700E^ and *Tet2* knock-out mice manifested a more severe anemia compared to mice with a sole alteration in *Sf3b1* or *Tet2* [12]. RNA-sequencing analysis of human and *Sf3b1*^+/K700E^ mice have shown that about 68% of splicing defects are due to alternative 3′SS usage occurring at −15 and −24 nucleotides upstream the canonical 3′ [12]. In vitro experiments expressing mutations often observed in human MDS in the yeast orthologue of *SF3B1* (Hsh155) have shown that those mutations alter the interaction of SF3B1 and Prp5 a protein helicase involved in the first ATP-dependent catalysis of the splicing cascade [25].

***U2AF1***. The U2-complex auxiliary factor 1 gene (*U2AF1*, 21q22.3) encodes a 35-kDa protein (alias *U2AF35*) of the U2-spliceosome responsible for recognition of the terminal 3′ AG dinucleotide in pre-messenger RNA introns. The protein has four major domains including two zinc finger regions, a serine-arginine (SR) domain, and an U2AF-homology domain. The U2AF1 unit forms a complex by heterodimerization with the 65-Kda protein called U2AF2 in order to bind the polypyrimidine tract upstream the 3′SS and recognize the branch point and the AG dinucleotide of the 3′SS. Mutations in *U2AF1* at codon S34 and Q157 are found in about 11% of patients with MDS and in 4% of patients with AML. Mutations are associated with a worse survival and are associated with an increased risk of AML transformation. *U2AF1* mutations result in the production of neomorphic phenotypes by chancing splicing patterns for many RNA downstream genes. These changes are lineage-specific and seem to influence the division of erythroid progenitors and subsequently change the differentiation trajectory of granulocytes and monocytes [26]. Analysis of the transcriptome of *U2AF1* mutant cells has shown changes in the splicing patterns (cassette exons) of genes present in the granulocytic compartment (*H2AFY*, *STRAP*) [27]. Primary cells expressing *U2AF1* mutations showed missplicing in mitotic (*ATR*, *CEP164*, *EHMT1*, *WAC*) and RNA processing (*PABPC4*, *PPWD1*, *PTBP1*, *STRAP*, *UPF3B*) genes [28]. Studies have also shown that cells expressing *U2AF1*^S34F^ have a decrease in ATG7 protein levels due to aberrant splicing resulting in a decrease in autophagy (as ATG7 is one of the key genes in autophagy). *U2AF1*^S34F^ cells with low levels of ATG7 and reduced autophagy had also increased reactive oxygen species and increased chromosomal instability [29]. Presence of *U2AF1* mutations was also correlated with increased IRAK4 isoforms which activate the NF-KB signaling pathway [30]. Genetically engineered murine models expressing *U2AF1*^S34F^ have been generated using the Cre recombinase tool and by activating Cre specifically in hematopoietic tissues. Upon Cre activation, mice expressing the S34 mutation developed features recapitulating MDS (dysplasia, cytopenias) as well as abnormal splicing profiles similar to the splicing patterns observed in human MDS [31]. Transgenic *U2AF1* mice also are sensitive to pharmacologic treatment with the splicing modulator, sudemycin [26].

***SRSF2.*** The *SRSF2* gene is located on chromosome 17q25.2 and encodes a member of the serine/arginine (SR)-rich family of pre-mRNA splicing components. It contains an RNA recognition motif (RRM) for binding RNA and an arginine and serine domain for binding other proteins. The arginine and serine domain is enriched in SR residues facilitating the interaction between SR and SF. SR proteins have versatile functions such as regulating pre-mRNA splicing, RNA stability, and translation. *SRSF2* mutations occur almost exclusively at proline 95 and alter binding affinity of the RRM motif. *SRSF2* mutations have been found in 28–47% of patients with CMML and about 14% of patients with MDS [32] and have been associated with increased age, higher levels of hemoglobin, and normal cytogenetics. *SRSF2* mutations are almost never sole mutations. In CMML, *SRSF2* mutations are often found with *TET2* mutations, while in AML are typically associated with *RUNX1*, *IDH2*, and *ASXL1* mutations. Pooled meta-analysis studies of MDS patients have shown that patients with *SRSF2* mutations predict for a worse survival and an increased risk for AML transformation [32,33] and have no prognostic effects in CMML [34].

In AML, *SRSF2* mutations have been found in about 25% of patients and associated with older age [35]. Studies have shown that proline 95 in normal SRSF2 forms a strong interaction with the second cytosine in the UCCAGU site and the second guanine in the UGGAGU site of the DNA. When mutations alter proline to histidine, the change in amino acid creates an alteration in the hydrogen bond leading to a change in the structural conformation. *SRSF2* mutations seem to induce differential splicing in *EZH2*, a known gene implicated in the pathogenesis of MDS and AML, and in several HNRNP proteins, including HNRNPA2B1, HNRNPH1, HNRNPM, and HNRNPH3 [36,37]. The diverse biological effects of *SRSF2* mutations are also demonstrated by the fact that other pathways are affected by the mutations, e.g., perturbation of double-strand DNA breaks, increase in p53 phosphorylation, and cell cycle arrest. In inducible Mx1-Cre *Srsf2*^+/P95H^ knock-in mice, sole alterations in *SRSF2* produce a phenotype similar to MDS. These features are also recapitulated by mice carrying homozygous *Srsf2* [36,38]

## 3. Other Splicing Factor Mutations

Mutations in other SF have been found in myeloid malignancies at lower frequencies and with patterns different from those of *SF3B1*, *SRSF2,* and *U2AF1*.

*ZRSR2* is a gene located on chromosome Xp22.2, which is mutated in about 5% of patients with MDS, predominantly males. The protein is another member of the SR-rich family of SF, and the gene encodes a component of the U2 auxiliary factor heterodimer which is responsible for the recognition of the 3′ splice acceptor site. The nature of the mutations resembles loss-of-function mutations [39]. Out-of-frame insertions and deletions, nonsense, missense, and splice site mutations have all been detected. No mutation hotspots have been observed and the mutations scatter across the entire coding region. In terms of splicing abnormalities, *ZRSR2* mutations cause abnormal splicing via intron retention of U12-depedent introns [39].

*PRPF8* is a gene located on chromosome 17p13.3 and has been found to be affected by somatic mutations or hemizygous deletions. A majority of patients (50%) with *PRPF8* mutations and del(17p) were found to be AML patients with poor prognosis. *PRPF8* alterations were found to correlate with increased RS and myeloblasts [40]. A subsequent analysis of a large cohort showed that *PRPF8* mutations were common and found in 4% (65/1700) of patients with MDS and AML [41]. Mutations were mainly missense, nonsense, frameshift, and splice site mutations, and showed a strong association with dismal prognosis. Based on studies of the yeast Prp8 protein, PRPF8 protein appears to be involved in spliceosome assembly, however a clear function in myeloid malignancies has not yet been demonstrated [42]. Cryo-electron microscopy technology has helped to resolve the structure of many components of the spliceosome and added more information on the function of PRPF8, specifically the N domain of PRPF8. PRPF8 interacts with U5 snRNA to stabilize the pre-mRNA in the spliceosome active site and it potentiates the U2 and U6 snRNA interactions with the intron [43].

*LUC7L2* is located on chromosome 7q34, and thus is a common target of gene deletions given the frequency of deletion of the long arm of chromosome 7 [del(7q)] and monosomy 7 (−7) in myeloid malignancies. The gene encodes a protein with a C2H2-type zinc finger, a coiled-coil region, and an SR domain. To date the function of LUC7L2 is not well characterized and the majority of what is known about the protein is based on observations of its ortholog splicing factor LUC7 which is involved in recruitment and interaction of SF. *LUC7L2* mutations can be hemizygous, heterozygous, and homozygous. *LUC7L2* mutations have been associated with shorter survival in patients with -7/del7q compared to patients with normal LUC7L2 expression [44,45]. *LUCL7L2* has been found aberrantly spliced in MDS cases harboring *SRSF2* small deletions [46]. Very rare mutations (<1%) have been found in other SF genes including *SF3A1*, *SF1*, *PRPF40B,* and *U2AF2* [5,47].

## 4. Therapeutic Intervention for Splicing Factor Mutations

As we have described, there are multiple ways by which pathologically altered splicing can promote the initiation and maintenance of cancer. In the last eight years, interest has grown in targeting splicing catalysis, splicing regulatory proteins, and specific altered splicing events. Splicing requires protein–protein and protein–RNA interaction, and is directed by a number of proteins, which are also subjected to regulation via post-translational modifications and protein–RNA interactions. This variety of interactions provides a range of possibilities to manipulate splicing for pharmacologic purposes. Pan-splicing modulators have been developed and are being tested in myeloid malignancies associated with SF mutations. Because splicing is ubiquitous and SF interact with many different proteins, it is possible that splicing modulators might also have effects against leukemic cells that do not harbor SF mutations [48]. Bacterially-derived products and their derivatives have been shown to bind the SF3B component to disrupt the early stages of the spliceosome cascade. These compounds have different stabilities in in vitro systems, and include low stability-agents such as FR901463, FR901464, FR901465, herboxidienes, and pladienolides, and high stability-agents such as E7107 (an analog of pladienolide B), spliceostatin A (SSA; from FR901464), and sudemycins, which block early spliceosome assembly [49]. While several compounds have only been shown to biologically alter splicing in vitro, a few compounds have been tested in both in vitro and in vivo. For instance, E7107 seems to induce marked splicing inhibition in several cellular and animal models [50]. The susceptibility of the spliceosomal mutant leukemia to splicing modulation via E7107 has also been validated in patient-derived xenografts obtained from primary AML cells with SF mutations [48].

Recently, H3B-8800 (a semi-derivative of pladienolides), a selective and orally bioavailable modulator of normal and mutant SF3b complex, has shown dose-dependent modulation of splicing in pre-clinical studies. Oral administration of H3B-8800 demonstrated preferential induction of cell growth arrest in several pre-clinical xenograft models of MDS/AML carrying SF mutations. H3B-8800 entered an open-label, multicenter phase 1 trial to evaluate pharmacokinetics/pharmacodynamics in AML, CMML, and MDS with SF mutations [51]. Several studies are ongoing using agents with non-spliceosome functions. More recently, several hematopoietic cells have been found more sensitive to the treatment with aryl sulfonamides (e.g., indisulam) compared to other cancer cell lines. Drug-sensitivity has been correlated with increased DCAF15 expression levels and copy number variation. The mechanism of action of indisulam seems to be shared with other sulfonamides such as tasisulam and chloroquinoxaline sulfonamide. Indeed, indisulam seems to induce the degradation of RBM39 (RNA binding motif protein 39) which in turn causes abnormal mRNA splicing (intron retention, exon skipping). RBM39 is a nuclear protein which associates with the E3 ubiquitin ligase complex (CUL4-DCAF15) in the presence of indisulam [52]. Inhibition of protein arginine methyltransferases (PRMTs) has also been correlated with increased anti-proliferative activity of SF mutant cell lines. The possibility of using protein arginine methyltransferases (PRMTs) inhibitors was demonstrated after subjecting murine AML cells driven by *MLL-AF9* fusion with and without *Srsf2*^P95H^ mutation to 45 compounds. *Srsf2* mutant cells were more sensitive than wild type cells to two PRMTs inhibitors, GSK591 (PRMT5 inhibitor) and MS023 (a pan type I PRMTs inhibitor) [53]. Very recently, *SF3B1* mutations have been shown to provoke interruption of the open reading frame of BRD9 with resultant decrease in the half-life of the protein. Cells derived from patients harboring *SF3B1* mutations have a reduced expression of BRD9 compared to cells of patients with wild type *SF3B1* [54].

Recently, luspatercept (ACE-536), a fusion protein containing a modified extracellular domain of the human activin receptor type IIB combined with a human IgG1 Fc domain and influencing the TGF-β and SMAD family proteins, is a phase II clinical trial for MDS patients and seems to induce a better clinical response in patients with *SF3B1* mutations [55]. In addition, *SF3B1* mutations represent a positive factor for erythroid response in a randomized phase II clinical trial of azacitidine and epoietin-β [56].

## 5. Conclusions

Mutations in SF genes are common in myeloid malignancies and induce different disease phenotypes according to cellular context. Each SF is also associated with a distinct pattern of mutations in other genes commonly detected in myeloid malignancies, as in the case of *SRSF2* mutations with *TET2* mutations in CMML and with *RUNX1*, *IDH2*, and *ASXL1* mutations in AML. Targeting SF mutations represents a novel avenue of drug discovery and development. In vitro studies have shown several different ways to impact splicing and restore splicing defects. Future experimental trials will shed light on the safety of pharmacologic agents targeting SF mutations.

## Abbreviations

**Gene Symbol**	**Official Gene Name**
*ABCB7*	ATP Binding Cassette Subfamily B Member 7
*ASXL1*	Additional Sex Combs Like 1
*ATR*	ATR Serine/Threonine Kinase
*BRD9*	Bromodomain Containing 9
*CEP164*	Centrosomal Protein 164
*DCAF15*	DDB1 And CUL4 Associated Factor 15
*DICER1*	Dicer 1, Ribonuclease III
*DNMT3A*	DNA Methyltransferase 3 Alpha
*EHMT1*	Euchromatic Histone Lysine Methyltransferase 1
*EZH2*	Enhancer Of Zeste 2 Polycomb Repressive Complex 2 Subunit
*HNRNPA2B1*	Heterogeneous Nuclear Ribonucleoprotein A2/B1
*HNRNPH1*	Heterogeneous Nuclear Ribonucleoprotein H1
*HNRNPH3*	Heterogeneous Nuclear Ribonucleoprotein H3
*HNRNPM*	Heterogeneous Nuclear Ribonucleoprotein M
*IDH1*	Isocitrate Dehydrogenase (NADP(+)) 1
*IDH2*	Isocitrate Dehydrogenase (NADP(+)) 2
*IRAK4*	Interleukin 1 Receptor Associated Kinase 4
*JAK2*	Janus Kinase 2
*LUC7L2*	LUC7 Like 2, Pre-MRNA Splicing Factor
*^¥^MACROH2A1*	MacroH2A.1 Histone
*MAP3K7*	Mitogen-Activated Protein Kinase Kinase Kinase 7
*NF1*	Neurofibromin 1
*PABPC4*	Poly(A) Binding Protein Cytoplasmic 4
*PDS5A*	PDS5 Cohesin Associated Factor A
*PML*	Promyelocytic Leukemia
*PPOX*	Protoporphyrinogen Oxidase
*PPP2R5A*	Protein Phosphatase 2 Regulatory Subunit B’Alpha
*PPWD1*	Peptidylprolyl Isomerase Domain And WD Repeat Containing 1
*PRMT*	Protein Arginine Methyltransferase
*PRPF40B*	Pre-MRNA Processing Factor 40 Homolog B
*PRPF8*	Pre-MRNA Processing Factor 8
*PTBP1*	Polypyrimidine Tract Binding Protein 1
*RBM39*	RNA Binding Motif Protein 39
*RUNX1*	RUNX Family Transcription Factor 1
*SF1*	Splicing Factor 1
*SF3A1*	Splicing Factor 3a Subunit 1
*SF3B1*	Splicing Factor 3b Subunit 1
*^≠^SF3B3*	Splicing Factor 3b Subunit 3
*SRSF2*	Serine And Arginine Rich Splicing Factor 2
*SRSF6*	Serine And Arginine Rich Splicing Factor 6
*STRAP*	Serine/Threonine Kinase Receptor Associated Protein
*TET2*	Tet Methylcytosine Dioxygenase 2
*TMEM14C*	Transmembrane Protein 14C
*U2AF1*	U2 Small Nuclear RNA Auxiliary Factor 1
*U2AF1L4*	U2 Small Nuclear RNA Auxiliary Factor 1 Like 4
*U2AF2*	U2 Small Nuclear RNA Auxiliary Factor 2
*UPF3B*	UPF3B Regulator Of Nonsense Mediated MRNA Decay
*WAC*	WW Domain Containing Adaptor With Coiled-Coil
*ZRSR2*	Zinc Finger CCCH-Type, RNA Binding Motif And Serine/Arginine Rich 2

Gene symbol and official names follow the nomenclature of the GeneCards [57]; ^≠^*SF3B3* alias SAP130 ^¥^*MACROH2A1* alias *H2AFY.*
